# Two Distinct Calmodulin Binding Sites in the Third Intracellular Loop and Carboxyl Tail of Angiotensin II (AT_1A_) Receptor

**DOI:** 10.1371/journal.pone.0065266

**Published:** 2013-06-05

**Authors:** Renwen Zhang, Zhijie Liu, Youxing Qu, Ying Xu, Qing Yang

**Affiliations:** 1 Department of Pathogenobiology, Bethune College of Medicine, Jilin University, Changchun, Jilin, P. R. China; 2 Computational Systems Biology, Department of Biochemistry and Molecular Biology, University of Georgia, Athens, Georgia, United States of America; University of Edinburgh, United Kingdom

## Abstract

In this study, we present data that support the presence of two distinct calmodulin binding sites within the angiotensin II receptor (AT_1A_), at juxtamembrane regions of the N-terminus of the third intracellular loop (i3, amino acids 214–231) and carboxyl tail of the receptor (ct, 302–317). We used bioluminescence resonance energy transfer assays to document interactions of calmodulin with the AT_1A_ holo-receptor and GST-fusion protein pull-downs to demonstrate that i3 and ct interact with calmodulin in a Ca^2+^-dependent fashion. The former is a 1–12 motif and the latter belongs to 1-5-10 calmodulin binding motif. The apparent Kd of calmodulin for i3 is 177.0±9.1 nM, and for ct is 79.4±7.9 nM as assessed by dansyl-calmodulin fluorescence. Replacement of the tryptophan (W219) for alanine in i3, and phenylalanine (F309 or F313) for alanine in ct reduced their binding affinities for calmodulin, as predicted by computer docking simulations. Exogenously applied calmodulin attenuated interactions between G protein βγ subunits and i3 and ct, somewhat more so for ct than i3. Mutations W219A, F309A, and F313A did not alter Gβγ binding, but reduced the ability of calmodulin to compete with Gβγ, suggesting that calmodulin and Gβγ have overlapping, but not identical, binding requirements for i3 and ct. Calmodulin interference with the Gβγ binding to i3 and ct regions of the AT_1A_ receptor strongly suggests that calmodulin plays critical roles in regulating Gβγ-dependent signaling of the receptor.

## Introduction

Angiotensin II (Ang II) plays crucial roles in the regulation of cardiovascular functions, all of which rely on signal propagation elicited by Ang II binding to G protein-coupled receptors (GPCRs). The most important Ang II receptor appears to be the AT_1_ receptor, which signals primarily through Gq/11 family G proteins, and which couples to Ca^2+^ mobilization, proliferation and/or hypertrophic signals in most cell types [1]. Despite intensive interest in the signal transduction pathways of the AT_1_ receptor, our understanding of many aspects of its signaling mechanisms is incomplete. Although most signals appear to be mediated through G proteins, other proteins can bind to and regulate AT_1_ receptor signal transduction [2].

Like other GPCRs, the heptahelical structure of the AT_1_ receptor consists of seven transmembrane α-helical barrels connected by three extracellular loops and three intracellular loops. The amino terminus is oriented extracellularly, whereas the carboxyl terminal tail faces the cytoplasm. The carboxyl terminal tail and the intracellular loops contain regulatory sites for G protein coupling, phosphorylation and protein-protein interactions. G proteins are thought to mediate signals through direct interaction of their α and/or βγ subunits with the intracellular domains of the GPCRs, generally at the juxtamembrane regions of the third intracellular loops (i3) and carboxyl terminus [3], [4], [5], [6], [7], [8].

There is a growing awareness that GPCRs signal by forming multimolecular complexes that include G proteins and many other proteins [9], [10], [11], [12], [13], [14]. In that regard, proteins other than G proteins have been shown to directly interact with the intracellular domains of GPCRs, primarily with the i3 loop and carboxyl tail. Binding of these so-called RIPs (receptor interacting proteins) can regulate receptor function, trafficking and signaling, either through, or independent of G proteins [2], [15], [16], [17]. For example, the β_2_ adrenergic receptor complexes with the Ca^2+^ channel Cav1.2, ensuring specificity and efficiency of signal propagation [9]. Thus, RIPs could be regulators for GPCRs [18], [19], [20], [21], could regulate signal propagation between GPCRs and their downstream binding partners [22], [23], and could possibly mediate signal propagation independent of G protein activation [10], [24]. Exploring RIPs and their binding sites in GPCRs could yield new clues for understanding signal propagation mechanisms of the GPCRs and developing therapeutic methods targeting their interactions.

Several putative RIPs for the 55 amino acid carboxyl terminal tail of the AT_1A_ receptor have been identified in recent years [10], [25], [26], [27]. In contrast, few putative RIPs have been reported yet for the relatively short (24 amino acids) i3 loop of the AT_1A_ receptor. One RIP that has drawn increasing interest of late is calmodulin (CaM), which is a small (≈ 17 kDa) acidic protein consisting of 148 residues. CaM has four EF-hand motifs with a Ca^2+^ binding site in each of the EF-hands. Binding of four Ca^2+^ ions to the EF hands elicits conformational changes that expose hydrophobic residues on the surface of CaM, enabling CaM to interact with its various target peptides. CaM recognition sequence motifs in proteins are highly variable, suggesting that features other than the primary sequence are critical for CaM binding. A typical CaM binding region is often characterized as a ≈20 amino acid α-helix with critical hydrophobic residues clustered on the opposite side of the helix from clusters of basic amino acid residues [28], [29].

Like many GPCRs, CaM participates in Ca^2+^ signaling; CaM can bind to and modulate the functions of enzymes, ion channels and receptors involved in a variety of cellular processes such as muscle contraction, cell cycle progression and cytoskeletal organization [30], [31], [32], [33]. Recently, CaM has been shown to directly interact with a handful of GPCRs, and to modify their functions. These include metabotropic glutamate receptor [34], [35], [36], [37], [38], [39], µ-opioid receptor [40], angiotensin II AT_1A_ receptor [41], D_2_ dopamin receptor [23], V2-vasopressin receptors [42], 5-HT_2A_ receptor [43] and 5-HT_2C_ receptor [44].

In that regard, one group used peptides and fusion proteins to identify a CaM binding site between residues 305–327 in the juxtamembrane region of the carboxyl terminal tail of the AT_1A_ receptor [41], although the functional significance of this interaction was not studied. The purpose of the current study was to identify and characterize CaM binding domain(s) in the AT_1A_ receptor, and to establish their functional significance. The work described in this manuscript supports the existence of two distinct CaM binding sites located in amino terminal juxtamembrane regions of the i3 loop and carboxyl tail of the AT_1A_ receptor. Moreover, the work supports a role for CaM as a regulator for signal propagation at the interface between AT_1A_ receptor and G protein βγ subunits.

## Materials and Methods

### Materials

GST expression vector pGEX-4T-1 and Glutathione-Sepharose 4B beads were purchased from Amersham Biosciences (Piscataway, NJ). The *E. coli* BL21 gold strain was purchased from Stratagene (La Jolla, CA). The yellow fluorescent protein fusion protein expression vector eYFP-N1 and the *Renilla* luciferase (RLuc) protein fusion protein expression vector were purchased from Clontech (Mountain View, CA). CaM and G protein Gβ antibodies were purchased from Upstate Biotechnology (Charlottesville, VA). Dansyl chloride, N-(6-aminohexyl)-5-chloro-1-naphthalenesulfonamide (W7), and antibiotic-antimycotic solution were purchased from Sigma-Aldrich (St. Louis, MO). Centricon YM-3 and YM-10 filters were purchased from Millipore (Billerica, MA). F-12 nutrient mixture, fetal bovine serum, trypsin-EDTA, isopropyl-β-D-thiogalactoside (IPTG) and neomycin (G418) were purchased from Invitrogen-GIBCO (Carlsbad, CA). Electrophoresis supplies and lipofectamine 2000 were purchased from Invitrogen (Carlsbad, CA). CaM and G protein βγ subunits purified from bovine brain, and protease inhibitor cocktail set I, were purchased from Calbiochem (La Jolla, CA). Crude peptides were synthesized at the Proteogenomics Facility of the Medical University of South Carolina (MUSC), and further HPLC purified at the Microchemical Facility at Emory University. The quality of the peptides was verified by mass spectrometric (MALDI) analysis. Oligonucleotide primers were synthesized by Integrated DNA Technologies, Inc. (Coralville, IA), and verified either by PAGE or HPLC depending upon the length of the primers.

### Cell Culture

Human Embryonic Kidney 293 (HEK293) cells from ATCC (Manassas, VA) were fed on a medium containing F-12 nutrient mixture, 1% antibiotic-antimycotic solution, and 10% fetal bovine serum. The cells were cultured at 37°C in a humidified incubator with 95% air and 5% CO_2_.

### Construction of GST-fusion Protein Expression Vectors

GST-ATi3(213–242) was synthesized by insertion of the PCR product of the i3 loop, spanning amino acids 213–242 of the AT_1a_R, into vector pGEX-4T-1 at *BamH I* and *EcoR I* enzyme sites downstream of GST, GST-TSYTLIWKALKKAYEIQKNKPRNDDIFRII. Forward and reverse primers were GGA TCC ACC AGC TAT ACC CTT ATT and GAA TTC AAT TAT CCT AAA GAT GTC with pcDNA3.1-AT_1A_ receptor as template. GST-ATi3N(213–234) encoding GST-TSYTLIWKALKKAYEIQKNKPR, was generated by introducing a stop codon into the vector of GST-ATi3(213–242) after the codon for 234R. Forward and reverse primers for generation of the stop codon were CAA AAG AAC AAA CCA AGA TAA GAT GAC ATC TTT AGG and CCT AAA GAT GTC ATC TTA TCT TGG TTT GTT CTT TTG. GST-ATi3C(235–242) was generated by insertion of a synthesized cDNA spanning amino acids 235–242 of AT_1A_ receptor encoding the peptide NDDIFRIT, into pGEX-4T-1. The complementary oligonucleotides GAT CCA ACG ATG ACA TCT TTA GGA TAA TTG and AAT TCA ATT ATC CTA AAG ATG TCA TCG TTG encoding the peptide were synthesized and annealed (500 mM Tris-HCL, pH 8.0, 100 mM MgCl_2_) at 90°C for 3 minutes, and then subcloned into pGEX-4T-1 at *BamH I* and *EcoR I* sites downstream of GST. GST-ATct(297–359) was constructed by same strategy as described for GST-ATi3(213–242). A cDNA spanning amino acids 297 to 359, (LNPLYFGFLGKKFKKYFLQLLKYIPPKAKSHSSLSTKMSTLSYRPSDNMSSSAKKPASCFEVE) was amplified by PCR from recombinant plasmid pcDNA3.1-AT_1A_ receptor. The PCR product was cloned into the pGEX-4T-1 vector at *BamH I* and *EcoR I* enzyme sites. Forward and reverse primers were GGA TCC CTG AAC CCT CTG TTC TAC and GAA TTC CTC CAC CTC AAA ACA AGA. GST-ATctN(297–324) encoding GST-LNPLFYGFLGKKFKKYFLQLLKYIPPKA, was constructed by introducing a stop codon after amino acid 324A into the vector of GST-ATct(297–359). The forward and reverse primers were CCCCCA AAG GCC TAG TCC CAC TCA AGC CTG TCT ACG, and CGT AGA CAG GCT TGA GTG GGA CTA GGC CTT TGG GGG. GST-ATctC(325–359) encoding GST-KSHSSLSTKMSTLSYRPSDNMSSSAKKPASCFEVE at the carboxyl terminus of the carboxyl tail of the AT_1A_ receptor, was constructed by the strategy used for construction of GST-ATi3(213–242), with forward primer GGA TCC AAG TCC CAC TCA AGC CTG and reverse primer GAA TTC CTC CAC CTC AAA ACA AGA. Fusion proteins encoding mutant receptor sequences GST-ATi3N(W219A), GSTATctN(F309A), GST-ATctN(F313A), GSTATi3(213–242)W219A, GST-ATct(297–359)F309A, and GST-ATct(297–359)F313A, were created with Stratagene’s Quick Change Mutagenesis Kit, using the corresponding non-mutant sequences as templates. Sequences of all of the constructs were verified by DNA sequence analysis in the MUSC Biotechnology Resource Laboratory.

### GST-fusion Protein Purification and Pulldown Assays

GST-fusion proteins were expressed in *E. coli* BL21 strain cells, and purified by affinity chromatography using immobilized glutathione sepharose 4B beads. Proteins were eluted by reduced glutathione in 50 mM Tris-HCl buffer (pH 8.0), and then concentrated with Centricon filters with 3 kDa MW cut-off. The eluted proteins were either used immediately or stored at −80°C for future use. For GST-fusion protein pull-down assays, fusion proteins were incubated with either cell lysates or purified protein(s) at 4°C for 3 hours, following which glutathione-sepharose 4B beads were added and incubation continued at 4°C for 1 hour. The beads were then recovered by brief centrifugation, followed by 4 washes with the corresponding incubation buffer. The fusion proteins and the proteins to which they were bound were then separated by SDS-PAGE, and identified by staining with Coomassie blue or by immunoblot.

### Measurements of Dansyl-CaM Fluorescence

Dansyl-CaM was synthesized according to a standard method [45], [46], [47], [48]. 500 µl of CaM purified from bovine brain (2 mg/ml in a buffer containing 20 mM NH_4_HCO_3_, pH 7.4 and 1 mM CaCl_2_) was mixed with 1 µl of dansyl chloride (100 mg/ml in acetone) at 4°C C for 1 hour with agitation. The mixture was then applied to a Centricon 4 concentrator with 10 kDa MW cut-off to remove the unincorporated dye. Efficiencies of dansylation (0.18–0.34 mol of dansyl moiety per mol of CaM) were calculated by measurement of absorption at 320 nm using a molar extinction coefficient of 3400 M-1cm-1. For conformational studies of dansyl-CaM, fluorescence was monitored at room temperature with a SLMAminco model 8000 series 2 spectrometer (Spectronic Instruments, Rochester, NY) at an excitation wavelength of 340 nm, and emission wavelengths from 425 to 600 nm.

### Structural Modeling of the CaM-peptide Complex

CaM-peptide complex structures were modeled based on the crystal structures of the bovine CaM-CaMKII peptide complex (PDB code 1 cdm [49]) and nematode CaM-cCaMKKp peptide complex (PDB code 1iq5 [50]). The CaM in PDB 1 cdm and liq5 share 100% sequence identity and very similar structures (0.8 Å backbone root mean square deviation between them), although they bind to different types of peptides (1-5-10 motif peptide CaMKII in 1 cdm and 1–16 motif peptide cCaMKKp in liq5). The coordinates of the 10 missing residues (74–83) of the CaM in 1 cdm were added through homology modeling based on 1iq5, and the resulting structure was further refined by 2000 steps of energy minimization using xplor-NIH [51].

#### CaM-ATct(302–317) modeling

The peptide CaMKII was manually mutated to ATct(302–317) in the molecular modeling software InsightII (Accelrys, Burlington, MA) based on the 1-5-10 motif alignment. This was accomplished by keeping the backbone structure intact while replacing the side-chains. The complex then was refined using 3000 steps of energy minimization using xplor-NIH. The F309A, Y312A, and F313A mutant structures were modeled based on the refined CaM-ATct structure through similar procedures.

#### CaM-ATi3(214–231) modeling

The peptide cCaMKKp was manually mutated to ATi3(214–231) in InsightII. This was done assuming an α-helical structure for ATi3(214–231). The first key motif residues in the two peptides (residue “1” in 1–16 and 1–12 motifs) were aligned, i.e., the first Leu in cCaMKKp was aligned to the first Leu in ATi3(214–231). Then, a protein docking procedure was conducted which minimizes the above CaMATi3(214–231) complex energy using a flexible protein-peptide docking algorithm [52]. The docking method used a Monte-Carlo annealing simulation and considered various movements including rigid peptide translation, rigid peptide rotation, peptide torsion on side-chain and backbone, and protein side-chain torsion. By keeping the protein backbone fixed, we considered both the flexibility of peptide and protein side-chain, in the docking simulation. 100 independent Monte-Carlo docking simulations were conducted from different random seeds. All of the obtained conformations were ranked using the same energy function used in the docking algorithm. The energy function includes atomic pair-wise interaction energy between protein and peptide and atomic solvation contribution. The conformation with the lowest energy was chosen and further refined using 3000 steps of energy minimization in xplor-NIH. The Y215A, L217A, and W219A mutant structures were modeled based on refined CaMATi3(214–231) structures in similar ways to those with the CaM-ATct (302–317) mutants. Evaluation of binding affinities: The binding affinities between CaM and the peptides were evaluated using the same energy function used in the docking program. Because the mutated residues were buried in the interface and the complex structures did not experience significant changes, the contribution of solvation energies was excluded. The final binding energy included the protein-ligand atomic pair-wise interactions, which were described by a distance-dependent function (Equation).
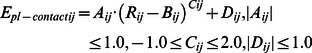



Rij is the actual distance between a pair of proteinligand atoms i and j, Aij is the force constant related to the atom pairs, Bij is a typical interaction distance between atoms of i and j, the exponent Cij determines the interaction’s distance dependence, and Dij corresponds to a basic packing background [52]. All units in the equation are in Å. All possible pair-wise contacts between protein atoms and peptide atoms within a contact cutoff of 15.0 Å were calculated, and the binding affinity was calculated as the sum of all of those interactions.

### Bioluminescence Resonance Energy Transfer (BRET)

BRET measures the transfer of energy between a donor luminescent source and an acceptor fluorophore. Our luminescent donor source was luciferase, whereas the acceptor fluorophore was yellow fluorescent protein, (YFP). When luciferase degrades its substrate coelenterazine, energy is released in the appropriate wavelength to excite YFP. The Forster energy transfer from luciferase to YFP only occurs when the donor and acceptor are <100 Å [53]. This means that the emission of light from YFP is a function of close physical proximity of the luciferase to the YFP. We used this method to demonstrate cellular interaction (close physical proximity) between the AT_1A_ receptor and CaM. In order to perform the BRET assays, we constructed expression vectors of AT_1A_ receptor-eYFP and RLuc-CaM (or CaM-RLuc), and co-transfected HEK293 cells with the two vectors. The AT_1A_ receptor-eYFP expression vector was constructed by insertion of the AT_1A_ receptor without stop codon into *Xho I* and *BamH I* sites at the N-terminus of eYFP and in frame with the eYFP in the eYFPN1 vector. Expression vectors of CaM-RLuc and RLuc-CaM were kindly provided by Dr. Justin Turner (Medical University of South Carolina), in which CaM was ligated in-frame with *Renilla* luciferase either at its N-terminus (CaM-RLuc) or C-terminus (RLuc-CaM) [43], [45].

We first stably transfected HEK293 cells with the AT_1A_ receptor-eYFP vector by using lipofectamine 2000 solution according to the vendor’s (Invitrogen) instructions. G418-selected colonies were then transiently transfected with either CaM-RLuc or RLuc-CaM fusion protein vectors by using the lipofectamine 2000 solution. The co-transfectants were detached with PBS buffer containing 1 mM EDTA, and distributed into 96 well plates at 10^5^ cells/well (OptiPlate 96, Greiner Bio-One. Longwood, FL). Fluorescence measurements were acquired using a Victor2 multilabel plate reader (Perkin Elmer Life Sciences, Shelton, CT). Coelenterazine was then added to a final concentration of 5 µM and sequential measurements were made with filters at 460±25 mm and 525±25 nm. The BRET ratio was calculated as the ratio of light emitted at 525 nM (YFP) over the light emitted at 460 nM (luciferease). The emission of light from YFP was measured both before and after stimulation with 100 nM of Ang II. Expression of AT_1A_ receptor-eYFP and RLuc-CaM (or CaM-RLuc) in HEK293 cells was verified by immunoblots using antibodies against YFP and RLuc.

### Statistical Analysis

Results were expressed as means ± S.E. ANOVA and student t-tests were used for the statistical analyses of the data.

## Results

### Interactions of CaM with the i3 Loop or Carboxyl Tail of the AT_1A_ Receptor

**Figure 1:**
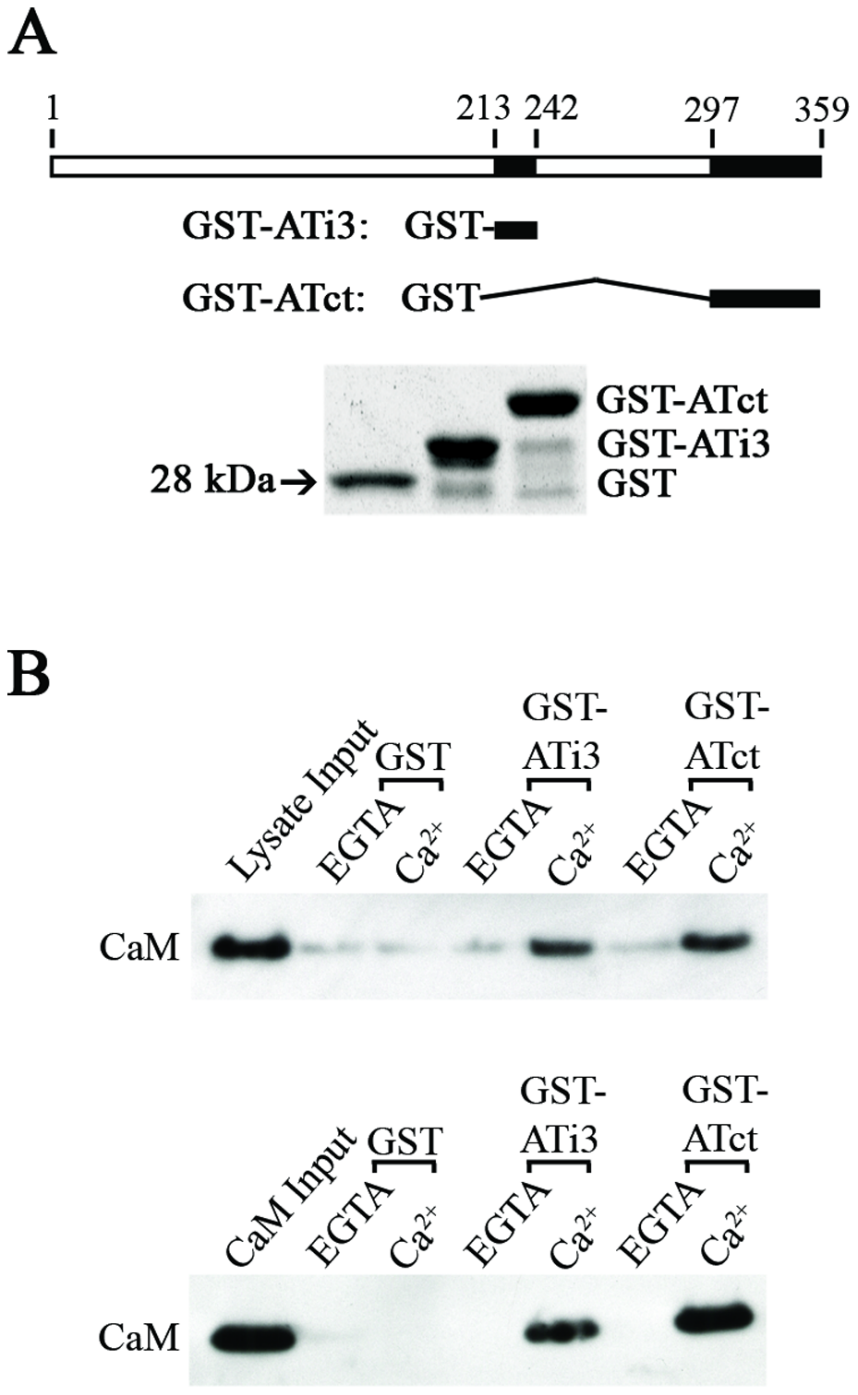
Interactions of the i3 loop and carboxyl tail of the AT_1A_ receptor with CaM. A, Construction of GST-fusion proteins. Upper panel illustrates a schematic structure of the AT_1A_ receptor and constructs of GST-fusion proteins of the i3 loop and carboxyl tail of the receptor. The numbers represent positions of amino acids in the receptor. The GST-fusion proteins were constructed, expressed in *E. coli*, purified by using gluthathione-sepharose 4B beads, separated by SDS-PAGE, and stained by Coomassie blue (lower panel). B. Interactions of the i3 loop or carboxyl tail of AT_1A_ receptor with CaM in rat brain lysates (upper panel) and with purified bovine brain CaM (lower panel). 50 pmol of GST-fusion proteins were incubated with 500 µg of rat brain lysates in 250 µl of buffer containing 20 mM Tris-HCl, 70 mM NaCl, pH 7.5 with 1 mM EGTA or 0.1 mM CaCl_2_. Interacting protein complexes were pulled down by gluthathione-sepharose 4B beads, and visualized by immunoblot with a specific anti-CaM antibody. The same methods were applied to interactions with pure bovine CaM (50 pmol), except that the buffer contained 100 mM Tris-HCl (pH 7.5). These experiments were repeated five times with similar results.

As an initial approach to identify interaction between CaM and the AT_1A_ receptor, we constructed expression vectors encoding GST fused to the i3 loop, GSTATi3(213–242), or to the carboxyl terminal tail, GST-ATct(297–359) of the AT_1A_ receptor. The vectors were introduced into *E. coli* (BL21 strain). Expression of GST-fusion proteins was induced by isopropyl-β-D-thiogalactoside (IPTG), following which the GST-fusion proteins were purified using gluthathione-sepharose 4B beads. The fusion proteins were separated by SDS-PAGE and visualized by Coomassie blue staining (Figure 1A). GST fusion protein pull-downs demonstrated that CaM associated with both the i3 loop and carboxyl terminal tail of the AT_1A_ receptor, in a Ca^2+^ dependent manner. Further, the interactions occurred not only with CaM from rat brain lysates (upper panel, Figure 1B) but also with pure bovine brain CaM (lower panel, Figure 1B), indicating that the interactions between the fusion proteins and CaM are direct.

### Interaction of CaM with the AT_1A_ Receptor in HEK293 Cells

**Figure 2:**
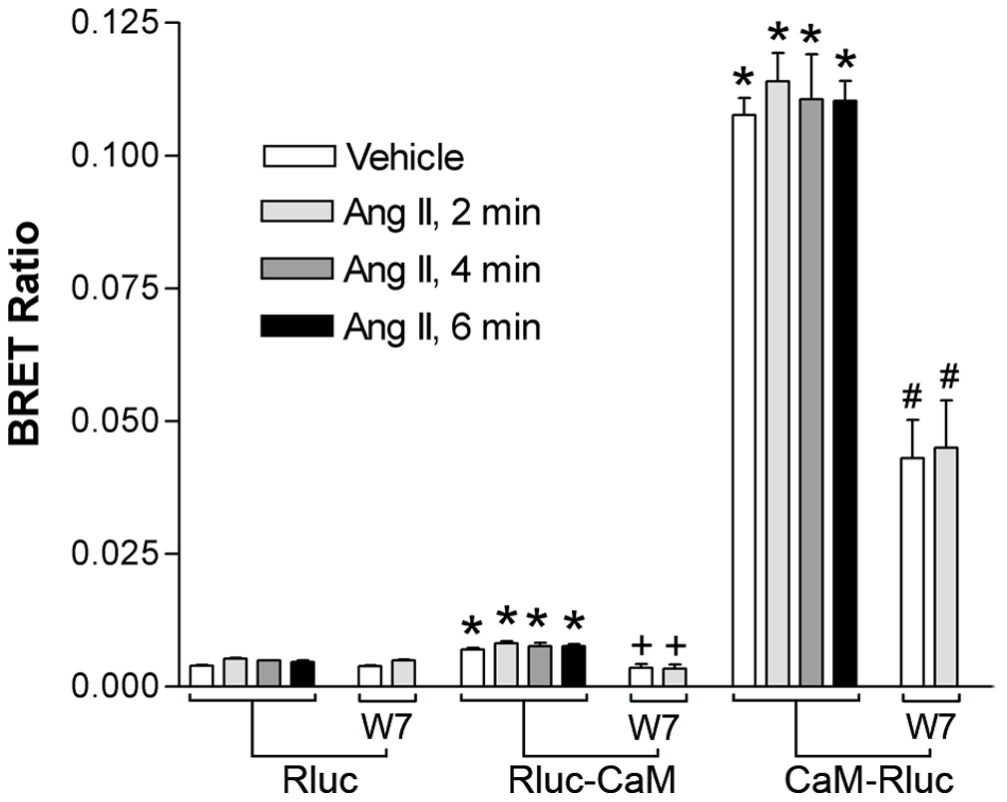
Interaction of the AT_1A_ receptor with CaM in HEK293 cells. HEK293 cells were stably transfected with an expression vector encoding AT_1A_ receptor-eYFP, following which cells were then transiently transfected with equal amounts of RLuc, RLuc-CaM or CaM-RLuc. 48 hours later, the cotransfectants were washed with saline pre-warmed at 37°C and calibrated in saline in a 37°C incubator for 30 minutes. Prior to BRET measurement, the co-transfectants were treated with CaM antagonist W7 (50 mM) for 20 minutes and/or of Ang II (100 nM ) for 6 min. Control co-transfectants were treated with equal amounts of saline (vehicle). BRET values were calculated as the ratio of light emitted at 525 nm over that at 460 nm. Results shown are mean ± S.E. of three separate experiments. **, ++ and ## stand for P<0.01 as compared with RLuc, RLuc-CaM or CaM-RLuc, respectively.

To test whether the interaction between the AT_1A_ holo-receptor and CaM also occurs in living cells, we employed a BRET assay. When HEK293 cells were co-transfected with expression vectors of both the AT_1A_ receptor fused with yellow fluorescent protein (AT_1A_ receptoreYFP), and CaM fused with luciferase either at its C-terminus (RLuc-CaM) or N-terminus (CaM-RLuc), the BRET ratio was significantly increased in cells co-transfected with YFP-AT_1A_ receptor and RLuc-CaM, and YFP-AT_1A_ receptor and CaM-RLuc, as compared to cells transfected with a control (RLuc). The BRET signals induced by YFP-AT_1A_ receptor+CaM-RLuc (26-fold) and YFP-AT_1A_ receptor+RLuc-CaM (0.75-fold) were markedly higher than that induced by co-transfection of YFP-AT_1A_ receptor and RLuc supporting a specific interaction between CaM and YFP-AT_1A_ receptor in that the position of the luciferase moiety on CaM influences the signal strength. Treatment of the transfected cells with a CaM inhibitor (W7) significantly reduced the BRET ratio to 51% of non-W7-treated YFP-AT_1A_ receptor+RLuc-CaM and 40% of non-W7 treated AT_1A_ receptor+CaM-RLuc transfectants. The effect of W7 appears not to be artificial as the BRET signal was not affected by W7 on RLuc transfectants, in which HEK293 cells were lacking RLuc-CaM or CaM-RLuc. These results provide further support for CaM-receptor interactions in that functional CaM is important for the BRET signal to occur. Interestingly, treatment of the co-transfectants with 100 nM Ang II at 2, 4 and 6 minutes did not alter the magnitude of the BRET ratio when compared with those without Ang II treatment (Figure 2). This suggests that the association between CaM and the AT_1A_ receptor is constitutive. If this is true, a complex containing AT_1A_ receptor and CaM could be pulled out from the cells.

### Potential CaM Binding Sites in the i3 Loop and Carboxyl Terminal Tail of the AT_1A_ Receptor

**Figure 3:**
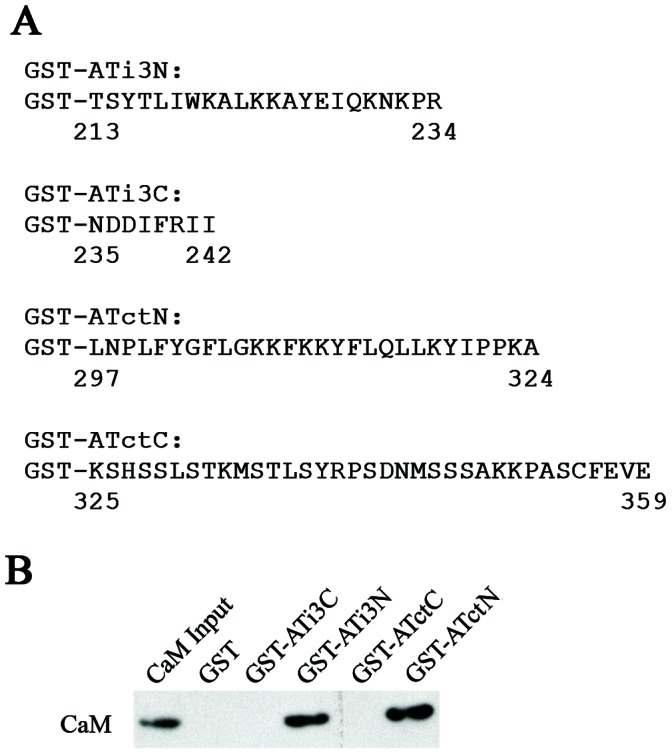
CaM binding sites in the i3 loop and carboxyl tail of the AT_1A_ receptor. A, Schematic representation of constructs of GST-fusion proteins. Four GST-fusion proteins containing truncated peptides in the i3 loop and carboxyl tail of the receptor were constructed, including GST-N-terminus of the i3 loop (GST-ATi3N), GST-C-terminus of the i3 loop (GST-ATi3C), GST-N-terminus of carboxyl tail (GST-ATctN) and GST-C-terminus of carboxyl tail (GST-ATctC). The numbers under the first or the last residues represent amino acid positions in the receptor. B, Interaction of GST-fusion proteins with CaM. The GST-fusion proteins (50 pmol) were incubated with purified bovine CaM (50 pmol) in 250 µl of buffer containing 100 mM Tris-HCl (pH 7.5) with 0.1 mM CaCl_2_. Proteins were pulled down by gluthathione-sepharose 4B beads, following which immunoblots were probed with a specific anti-CaM antibody. These experiments were repeated five times with similar results.

In order to identify potential CaM binding sites in the AT_1A_ receptor, we searched the Calmodulin Target Database at the Ontario Cancer Institute (http://calcium.uhnres.utoronto.ca/ctdb/flash.htm). The database evaluates amino acid sequences for the presence of characteristics associated with known CaM binding sites. Using this algorithm, two putative CaM binding sites were identified in the N-terminal juxtamembrane region of the i3 loop spanning amino acids from 210 to 234, and in the juxtamembrane area of the carboxyl tail spanning amino acids from 297 to 324. Accordingly, we constructed four GST fusion proteins containing the N- or C-terminal portions of both the i3 loop and the carboxyl tail (Figure 3A). These were utilized to document potential interactions with CaM by GST-fusion protein pull-down assays. Results of those experiments showed that the N-terminal sequences in the i3 loop ATi3N (213–234) and the carboxyl tail ATctN (297–324) interact with purified CaM. In contrast, the C-terminal sequences in the i3 loop, ATi3C (235–242), and in the carboxyl tail, ATctC (325–359), had no interactions with CaM (Figure 3B). The experimental data agree with the theoretical predictions from the CaM Target Database in that the predicted CaM binding domains associate with CaM, whereas nearby sequences do not.

### Recognition of CaM Binding Motifs in the i3 Loop and Carboxyl Tail of the AT_1A_ Receptor

**Figure 4:**
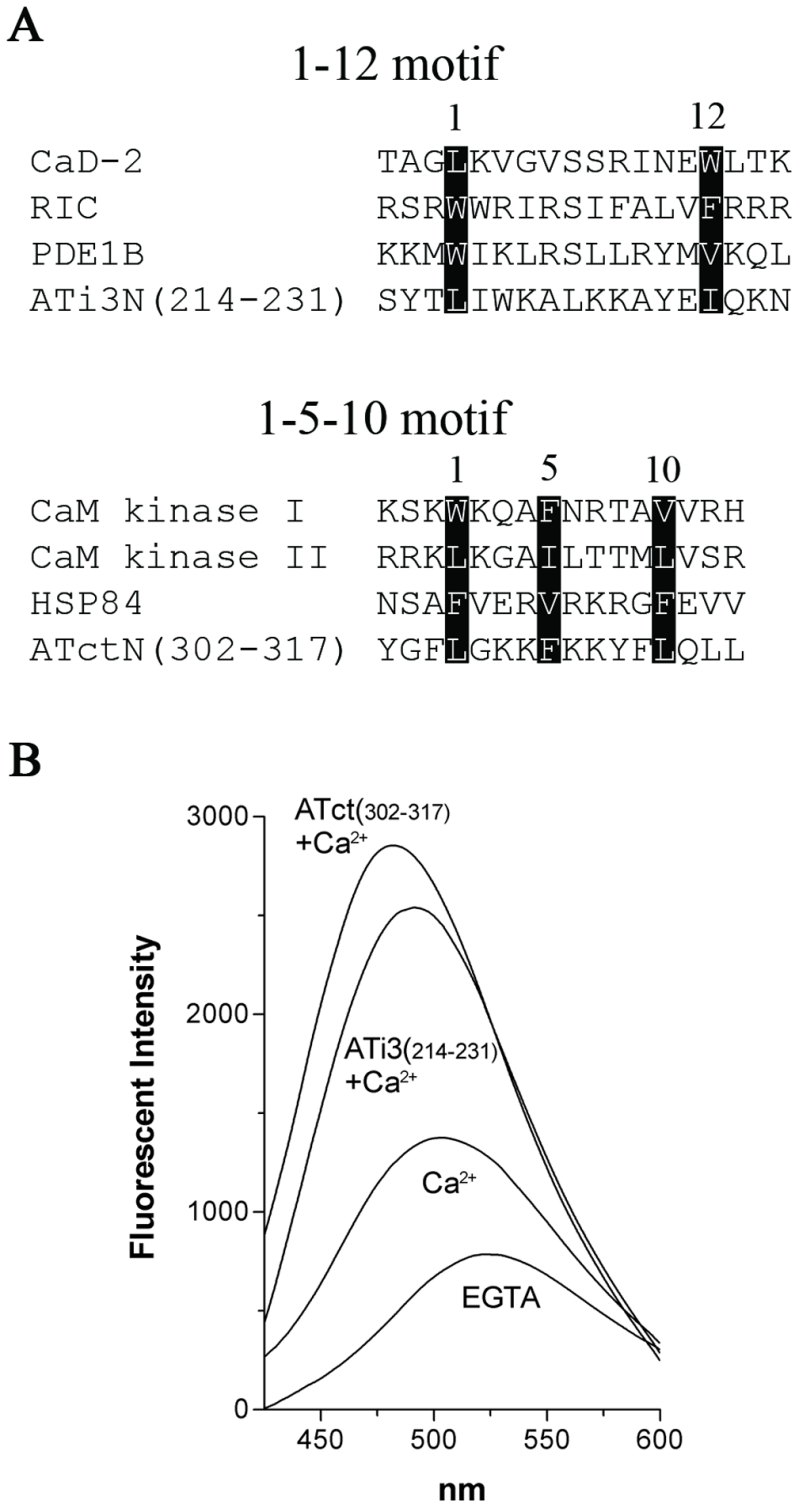
CaM binding motifs in the i3 loop and carboxyl terminal tail of the AT_1A_ receptor. A, Alignment of CaM binding motifs. Based on conservative hydrophobic amino acid distributions (the highlighted amino acids), the N-terminus of the i3 loop of receptor possesses a 1- 12 CaM binding motif, and the N-terminus of the carboxyl terminal tail of the receptor possesses a 1-5-10 CaM binding motif. The abbreviations are: CaD for caldesmon (CaD)-2, RIC for Ras, which interacts with calmodulin, PDE1B for phosphodiesterase 1B, and HSP84 for heat shock protein (human). Calmodulin kinase I and calmodulin kinase II are the bovine brain form and rat γ form, respectively. B, CaM interaction with CaM binding motifs in the i3 loop and carboxyl tail of the receptor. Fluorescence emission spectra of dansyl-CaM (106 nM) at an emission wavelength of 425–600 nm were measured in the presence of 10 mM EGTA, 0.1 mM calcium, 500 nM ATi3 (214–231) +0.1 mM calcium, and 500 nM ATct (302–317) +0.1 mM calcium. The measurements were performed after incubation for 1 hour at room temperature. Spectra were corrected for background buffer fluorescence. The curves are averaged from three separate experiments.

Alignment of the sequences of the ATi3N and the ATctN with known CaM interacting motifs indicated that ATi3N possesses a 1–12 motif S214YTLIWKALKKAYEIQKN231, and AtctN possesses a 1-5-10 motif Y302GFLGKKFKKYFLQLL317 (Figure 4A). The main feature of the 1–12 motif is the location of two hydrophobic residues (FILVW) at positions 1 and 12, and of the 1-5-10 motif is that two hydrophobic residues are separated by eight amino acids with one hydrophobic amino acid anchored in the middle. To experimentally test the CaM binding motifs in the AT_1A_ receptor, we measured shifts in the fluorescence emission spectrum of dansyl-CaM as a means to detect major conformational alterations in CaM. In that regard, the fluorescence emission spectrum of dansyl-CaM was markedly shifted by addition of Ca^2+^ (λmax from 521 to 506 nm), with about a 1.7 fold increase in fluorescence intensity at 506 nm, when compared with dansyl-CaM in the presence of the Ca^2+^- chelating agent, EGTA. In the presence of Ca^2+^, a synthetic peptide containing the motif ATi3(214–231) further shifted the spectrum (λmax from 521 to 486 nm), with about a 3.2 fold increase in fluorescence intensity at 486 nm. A synthetic peptide containing the motif ATct(302–317) also markedly shifted the spectrum (λmax from 521 to 482 nm), with about a 3.5 fold increase in fluorescence intensity at 482 nm (Figure 4B). These data indicate that upon loading of CaM with Ca^2+^ ions, a conformational change in CaM is induced, and this conformational shift is markedly enhanced by binding of CaM to ATi3(214–231) or ATct (302–317). These data further support the potential interactions of the putative CaM binding regions of the AT_1A_ receptor with CaM.

### Effect of Point Mutations on CaM Binding in the i3 Loop and Carboxyl Tail of the AT_1A_ Receptor

**Figure 5:**
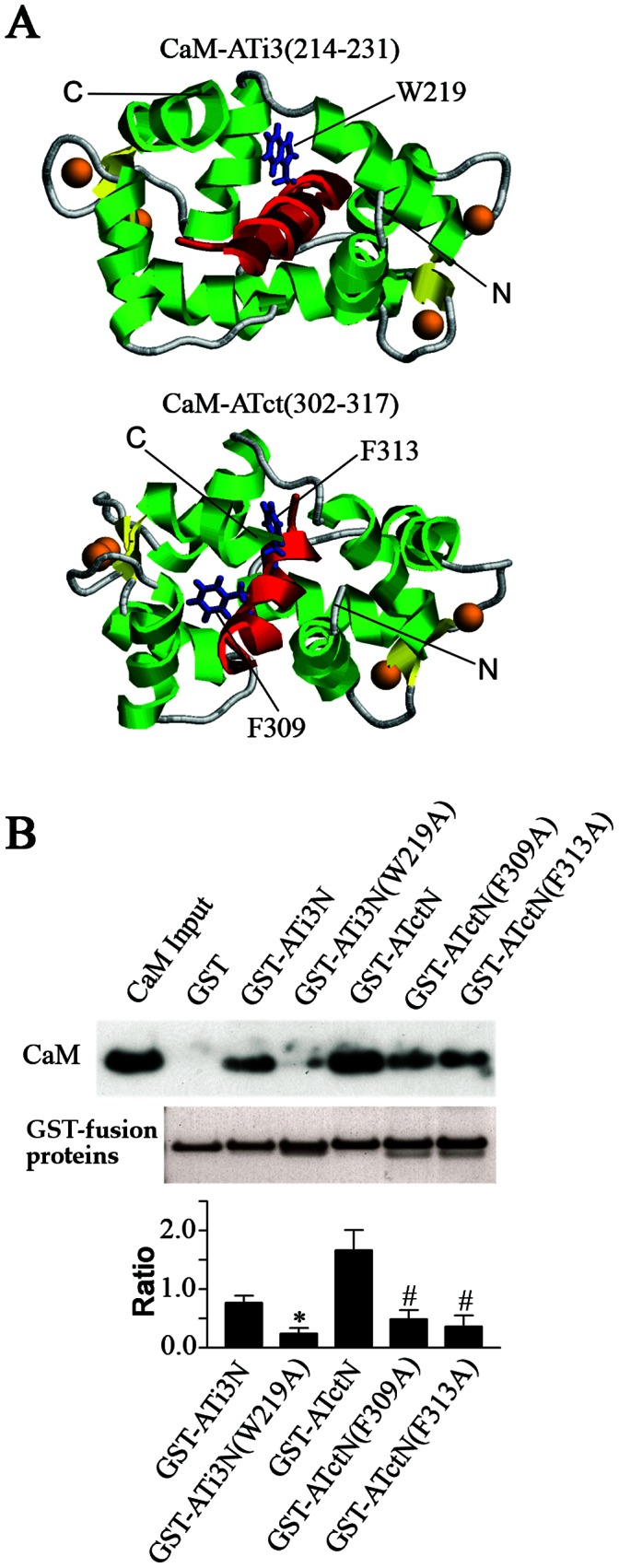
Effect of point mutations in ATi3N or ATctN on their interactions with CaM. A. Modeled structures of CaM-ATi3(214–231) and CaM-ATct(302–317). The complexes of CaM and CaM binding motif in the i3 loop ATi3(214–231) or the carboxyl terminal tail ATct(302–317) of the receptor were modeled as described in Experimental Procedures. The target peptides are colored in red. Residues W219 in ATi3(214–231)−SYTLIWKALKKAYEIQKN, and F309 and F313 in ATct(302–317)−YGFLGKKFKKYFLQLL are displayed with sticks and are colored in blue. Calcium atoms are shown as orange spheres. The N- and C- termini of CaM are also labeled. Helices and sheets in CaM are colored in green and yellow, respectively. B. Effect of point mutations at ATi3N or ATctN on their interaction with CaM. 50 pmol of wild type GST-fusion proteins including GST-ATi3N(213–234) and GST-ATctN (297–324), and 50 pmol of mutated GST-fusion proteins including GST-ATi3N(W219A), GST-ATctN(F309A) and GST-ATctN(F313A), were incubated with purified bovine brain CaM in a buffer containing 100 mM Tris-HCl (pH 7.5) with 0.1 mM CaCl_2_. The protein complexes were pulled down by gluthathione-sepharose 4B beads, and subjected to immunoblot with a specific anti-CaM antibody. GST-fusion proteins were visualized in the gels by Coomassie blue staining (the lower gel panel). The summary graph represents relative densities of the ratio of the CaM in the immunoblots and the loaded GST-fusion proteins as determined by Coomassie blue staining. The bars represent mean ± S.E. from 5 independent experiments. * or # stand for P<0.01 as compared with wild type GST-fusion proteins, respectively.

If the interactions between the AT_1A_ receptor peptides and CaM are specific, we would expect that mutations of key residues in each of the peptides would reduce the interactions between CaM and the peptides. We modeled three-dimensional complex structures of CaM bound to ATi3(214–231) and to ATct(302–317) in order to predict amino acid changes that could reduce the interactions between the i3 loop or the carboxyl tail of AT_1A_ receptor and CaM (Figure 5A). Based on non-bonded contact analysis on the CaM-peptide complex structures using the HBPLUS program [54], we selected for alanine mutagenesis, six residues within the motifs at predicted contact sites with CaM. The residues chosen for study based on contact site proximity were Y215A, L217A, and W219A located within the i3 loop, and F309A, Y312A, and F313A located within the carboxyl tail. We used the modeled complexes described in Figure 5A to determine the theoretical binding energies of each mutant peptide. The calculated binding energies were higher than wild type CaM-ATi3(214–231) for CaM-ATi3(214–231)W219A (+8%), and lower for CaMATi3(214–231)Y215A (-12%) and CaMATi3(214–231)L217A (-1%), respectively. The calculated binding energies were found to be higher than wild type CaM-ATct(302–317) for CaM-ATct(302–317)F309A (+7%) and CaMATct(302–317)F313A (+10%), and lower for CaM-ATct(302–317)Y312A (-1%). Because higher binding energies correspond to lower affinities, we predicted that GST fusion proteins bearing W219A, F309A and F313A would have reduced efficiencies in pulling down recombinant CaM. In that regard, we performed GST-fusion protein pull-down assays with fixed concentrations of AT_1A_ receptor peptide fusion proteins and CaM to determine relative binding efficiencies. Those studies demonstrated that the relative binding efficiencies for CaM are significantly reduced in the three mutants predicted to have a higher binding energy, GSTATi3N(213–234)W219A (32% of wild type), GST-ATctN(297–324)F309A (29% of wild type) and GST-ATctN(297–324)F313A (22% of wild type) (Figure 5B). Indeed, mutants GST-ATi3N(213–234)Y215A, GST-ATi3N(213–234)L217A and GST-ATctN(297–324)Y312A did not have reduced binding efficiencies (not shown), so those mutants were not further studied.

### Titration of Dansyl-CaM Fluorescence Changes with Peptides

**Figure 6:**
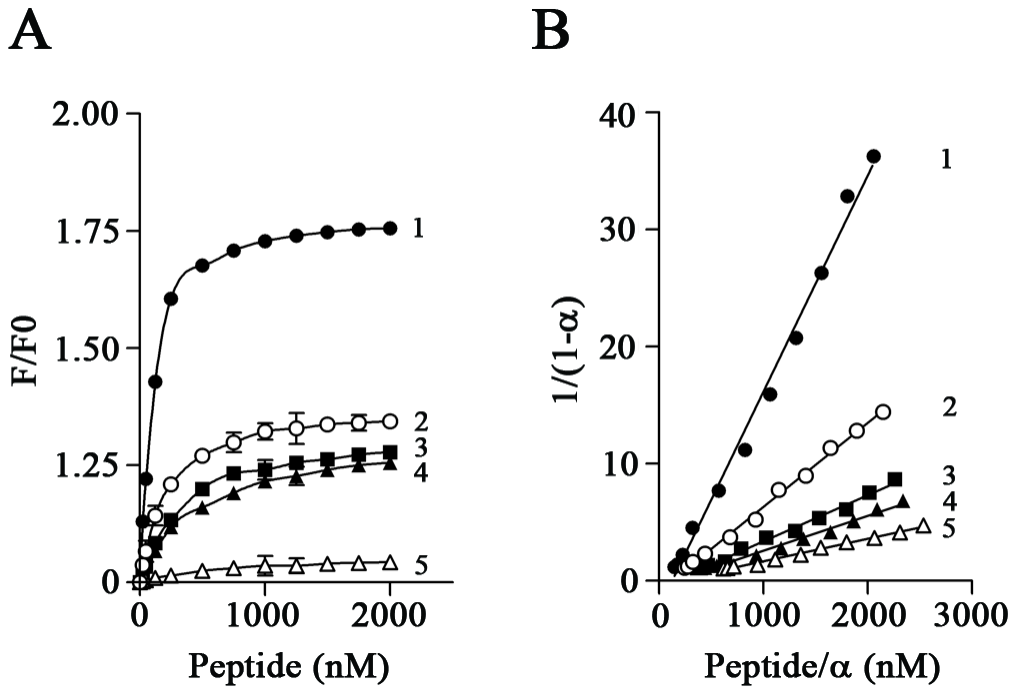
Titration of dansyl-CaM fluorescence with peptides ATct(302–317)(1), ATi3(214–231)(2), ATct(302–317)F309A(3), ATct(302–317)F313A(4), and ATi3(214–231)W219A(5). Peptides (25–2000 nM) were incubated with 147 nM dansyl-CaM in the presence of 0.1 mM calcium for 1 hour at room temperature. Fluorescence emission of dansyl-CaM was then measured at an emission wavelength of 485 nm. The relative fluorescence intensities F/F0 (the ratio of the total fluorescence intensities and the fluorescence intensity of dansyl-CaM) were plotted against the concentration of the peptides added (curve A), which represents an average of three separate experiments (mean ± S.E.). The curves in B were derived from calculation of the titration data according to a previously described method [58]. The α representing the fractional degree of saturation of dansyl-CaM fluorescence is calculated from formula α = (F−F0)/(F∞−F0) where F∞ is the fluorescence intensity at the saturating level of the peptides added. The reciprocal of the slope gives the apparent dissociation constants (Kd) of CaM for the peptides, which are: 79.4±7.9 nM for ATct (302–317), 177.0±9.1 nM for ATi3 (214–231), 308.1±11.8 nM for ATct(302–317)F309A, 388.1±9.4 nM for ATct(302–317)F313A, and 587.7±13.6 nM for ATi3(214–231)W219A (data are expressed as mean ± SE).

We next determined the binding affinities of CaM for peptides ATct(302–317), ATi3(214–231), ATct(302–317)F309A, ATct(302–317)F313A, and ATi3(214–231)W219A by titration of dansyl-CaM fluorescence in the presence of increasing concentrations of the peptides. The apparent dissociation constants (Kd) of CaM for the peptides were 79.4±7.9 nM for ATct(302–317), 177.0±9.1 for ATi3 (214–231), 308.1±11.8 for ATct(302–317)F309A, 388.1±9.4 for ATct(302–317)F313A, and 587.7±13.6 for ATi3(214–231)W219A (Figure 6).

### CaM Inhibits the Interaction between G Protein βγ Subunit and the AT_1A_ Receptor

**Figure 7:**
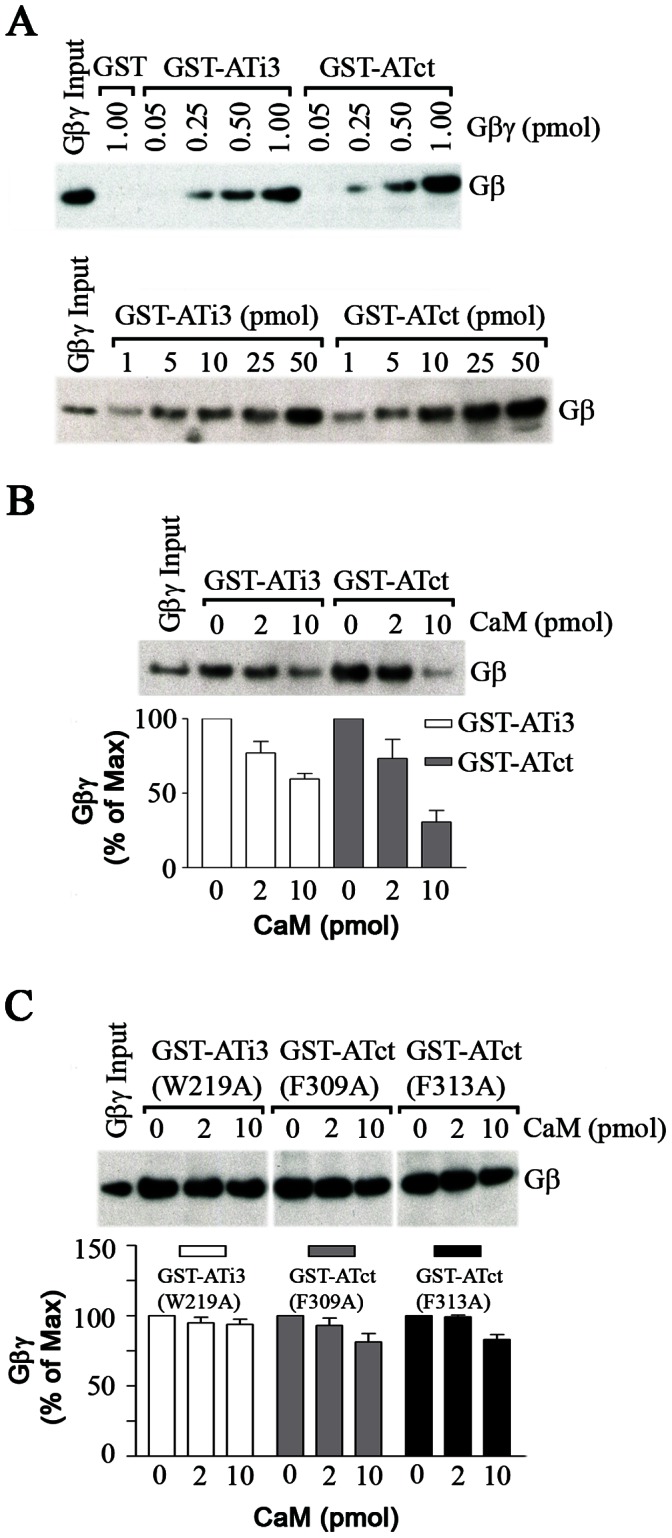
Effect of CaM on G protein βγ subunit interaction with wild ATi3 or ATct. A. Interaction of G protein βγ subunits and ATi3 or ATct. 50 pmol of GST-ATi3(213–242) or GST-ATct(297–359) were incubated with different amounts of G protein Gβγ subunits in a buffer containing 20 mM Tris-HCl and 70 mM NaCl (pH 7.5). Interactions were assessed by GST-fusion protein pull-down assay, and blots were probed with a specific antibody against Gβ subunits (upper panel). 1 pmol of Gβγ subunit was interacted with different amounts of GST-ATi3(213–242) and GST-ATct(297–359). Interaction was assessed by immunoblot against Gβ subunits (lower panel). B. CaM inhibits the interaction between G protein Gβγ subunits and ATi3 or ATct. 2 pmol of GST-ATi3(213–242) or GST-ATct(297–359) were incubated with different concentrations of pure bovine brain CaM for 30 minutes in a buffer (20 mM Tris-HCl and 70 mM NaCl with 0.1 mM CaCl_2_, pH 7.5) following which 2 pmol of Gβγ subunits were added and incubated for 1 hour. GST-fusion proteins and their interacting proteins were pulled down by gluthathione-sepharose 4B beads, and subjected to immunoblot. Blots were probed with a specific antibody against Gβ subunits. The summary graph represents means ± S.E. from four independent experiments. C. Effects of CaM on the interactions between G protein βγ subunits and mutated ATi3 and ATct. The method is same as the described in the Figure 7B, except that we used mutated GST-fusion proteins, GST-ATi3(213–242)W219A, ATct(297–359)F309A, and ATct(297–359)F313A. The summary graph represents mean ± S.E. from 4 or 5 independent experiments.

We wanted to investigate potential roles for CaM interaction with the AT_1A_ receptor. One possibility is that CaM could modulate the coupling of the receptor to G proteins. In that regard, G protein βγ subunits bind to the i3 loop and carboxyl tail of AT_1A_ receptor in a concentration-dependent fashion (Figure 7A). Interestingly, Figure 7B demonstrates that the interactions between Gβγ subunits and the i3 loop or the carboxyl tail can be inhibited by CaM. The interaction of 2 pmol of Gβγ with 2 pmol of GST-i3 loop was significantly reduced by 2 and 10 pmol of CaM to 77% and 60%, respectively. The interaction of 2 pmol of Gβγ with 2 pmol of GST-ct was significantly reduced by 2 and 10 pmol of CaM to 73% and 34%, respectively (Figure 7B).

In order to confirm that the inhibitory effects of CaM on binding of Gβγ subunit to the ATi3 loop and ATct are specific, we performed similar studies using GST fusion proteins that incorporated the same mutations described in Figure 6 (W219A), F309A, and F313A). Figure 7C demonstrates that all three mutant fusion proteins could effectively bind Gβγ subunits. The binding for all three mutants was similar in magnitude to the binding to non-mutated ATi3 and ATct fusion proteins (not shown). These data suggest that mutations that decrease CaM binding to the AT_1A_ receptor i3 loop and carboxyl terminal tail have no effect on Gβγ binding. Further, CaM had no significant effects on Gβγ binding to the three mutant fusion proteins. In the presence of 2 and 10 pmol of CaM, the amount of Gβγ binding to GST-ATi3(W219A) was 95%, and 94%, to GSTATct(F309A) was 93% and 81%, and to GSTATct(F313A) was 99%, and 83%, respectively (Figure 7C). Thus, these experiments document three key points: (1) Both Gβγ and CaM bind to GST-ATi3 and GST-ATct. (2) CaM can impair binding of Gβγ to GST-ATi3 and possibly more so to GST-ATct. (3) Binding of CaM to GST-ATi3 and GST-ATct is required for impairment of Gβγ binding.

## Discussion

What is new about this work is that (1) we have identified and characterized two distinct CaM binding motifs in the AT_1A_ receptor, one each in the amino terminal juxtamembrane regions of the i3 loop and carboxyl terminal tail of the AT_1A_ receptor, and (2) we have demonstrated that CaM impairs the binding of Gβγ to GST-fusion proteins containing each of those two sequences from the AT_1A_ receptor. These findings suggest that direct binding of CaM to intracellular regions of the AT_1A_ receptor inhibits Gβγ binding, and can modulate its signaling. Thus, CaM binding to the AT_1A_ receptor is functionally significant.

In this manuscript, we have presented multiple lines of evidence supporting the existence of CaM-binding domains within the angiotensin AT_1A_ receptor. (1) The AT_1A_ receptor contains two putative CaM-binding domains as identified by a computer search algorithm and by molecular modeling studies. (2) GST-fusion proteins encompassing the AT_1A_ receptor i3 loop and carboxyl terminal tail efficiently pulled down CaM from rat brain lysates and from solutions of purified bovine CaM, and those interactions were Ca^2+−^dependent. (3) BRET studies demonstrated that CaM and the AT_1A_ holo-receptor are in close proximity (within the Forster radius) when transfected into HEK293 cells, suggesting that the interaction can occur in intact cells. The BRET signal was significantly diminished by a CaM inhibitor W7, suggesting that functional CaM is required for the BRET signal to be generated. The association of CaM and the AT_1A_ receptor was not affected by agonist stimulation, suggesting that CaM and the receptor could form a constitutive complex, as has already been shown for the 5-HT_1A_ receptor [45]. (4) Mapping studies showed that the amino terminal juxtamembrane regions of the AT_1A_ receptor i3 loop and carboxyl terminus interact specifically with CaM as determined by GST pull down assays and by dansyl-CaM fluorescence spectral measurements. (5) Exogenously applied CaM significantly diminished binding of purified Gβγ subunits to GST fusion proteins containing the putative CaM binding regions in the AT_1A_ receptor i3 loop and carboxyl terminus. Fusion proteins containing point mutations of the CaM binding domains that significantly reduce affinities for CaM had no effect on binding of purified Gβγ subunits to the fusion proteins, but significantly attenuated the ability of CaM to diminish binding of purified Gβγ subunits. The latter results suggest that CaM binding to the CaM binding domains is required for CaM to effectively diminish Gβγ subunit interaction with the AT_1A_ receptor i3 loop and carboxyl terminus.

We used a combination of techniques including GST-fusion protein pull-downs, site-directed mutagenesis, and dansyl-CaM fluorescence to pinpoint the amino terminal juxtamembrane regions of the AT_1A_ receptor i3 loop and carboxyl terminus of the receptor as the CaM binding domains. In that regard, truncated portions of the i3 loop (amino acids 213–234) and carboxyl tail (residues 297–324) were able to efficiently pull-down CaM, whereas adjacent regions of the receptor were not. Alignment of the truncated sequences with other well established CaM binding motifs suggested that the two CaM binding motifs in the AT_1A_ receptor could be narrowed down to amino acids 214–231 in the i3 loop, and 302–317 in the carboxyl tail. Our work is consistent with that of Thomas and colleagues, who previously identified a putative CaM binding domain in a fusion protein from the juxtamembrane region of the AT_1A_ receptor carboxyl terminus [41]. Furthermore, the interaction appeared to be of high affinity in that the peptide induced a CaM mobility shift in a urea gel, which is generally indicative of an affinity better than 100 nM. Our work validates and extends their report in a number of respects. We showed that the AT_1A_ holo-receptor interacts with CaM by the BRET method. We identified a second CaM interaction domain in the i3 loop of the AT_1A_ receptor, identified CaM interaction motifs and calculated the affinities of the interaction of CaM for both sites. We identified three residues in the AT_1A_ receptor (W219, F309, and F313) that are critical for efficient coupling of CaM to the receptor, by computer modeling and mutagenesis. We also showed that CaM binding is necessary to inhibit binding of G protein βγ dimers to both sites. Moreover, the binding requirements for βγ and CaM are distinct in that mutations that reduced the binding of CaM to both regions had little measurable effect on βγ binding.

We measured the affinities of synthetic peptides corresponding to those regions by examining the spectral shifts of dansyl-CaM in the presence of Ca^2+^ and candidate peptides, assuming that spectral shifts reflect induction of dramatic conformational changes in CaM. Indeed, it is known that CaM assumes a dumbbell-shaped conformation upon loading with Ca2+ [55], [56], and a compact globular conformation when interacting with various target peptides [49]. Both putative CaM binding domains were shown to have relatively high affinities for CaM, 79.4±7.9 nM for ATct(302–317), and 177.0±9.1 for ATi3(214–231). The differences in the affinities of these peptides for CaM could be functionally significant in that CaM appears to be somewhat more effective in diminishing Gβγ binding to the AT_1A_ receptor carboxyl terminus than to the i3 loop (Figure 7B). These affinities are similar to those reported for other GPCRs, including the 5-HT_1A_ (87 nM and 1.70µM.) and 5-HT_2A_ (65 and 168 nM) receptors.

CaM is an important regulatory molecule, which functions as the major calcium-sensor in most cells [30]. CaM has been shown to regulate many signaling molecules and effectors, including kinases, phosphatases and other enzymes, ion channels, transcription factors, receptors and cytoskeletal proteins. CaM has also been shown to bind to a small number of GPCRs, although the functional significance of these interactions is only now being elucidated. CaM binding to D_2_-dopamine, 5-HT_1A_, μ-opioid and group III metabotropic glutamate mGluR7a receptors, regulates functional coupling of the receptors to pertussis toxin-sensitive heterotrimeric Gi/o protein α-subunits [36], [40], [45], [57], [58]. Similarly, CaM binding to the 5-HT_2A_ and V_2_ vasopressin receptors attenuates coupling to Gq/11 α-subunits, GTPγS binding and/or Ca^2+^ mobilization [42], [43]. CaM binding also impairs phosphorylation of peptides derived from regulatory regions of the 5-HT_1A_, 5-HT_2A_, and mGluR5 receptors [37], [43], [45]. Thus, CaM can attenuate both receptor phosphorylation and propagation of G protein a-subunit-dependent signals for a small group of GPCRs.

A role for CaM in regulating Gβγ subunit binding to metabotropic glutamate receptors has previously been proposed. O’Connor and colleagues showed that CaM can bind to a fusion protein containing the carboxyl terminus of the metabotropic glutamate receptor subtype 7 (mGluR7), and that CaM inhibited βγ binding to the same sequence [34]. The authors suggested that G protein-mediated signaling was enhanced by CaM through displacement of Gβγ subunits from the carboxyl terminus. El Far and colleagues confirmed those observations, and described similar motifs in the carboxyl termini of mGluR4A, mGluR7B, mGluR8A, and mGluR8B, perhaps indicating that the mutually exclusive binding of CaM and Gβγ to the carboxyl termini of mGluR is an important signaling mechanism [36]. Up to now, this effect has only been described for mGluRs, which are class 3 GPCRs [59]. Our work demonstrates that this process is not limited to mGluRs or class 3 GPCRs, in that it occurs in the carboxyl terminus of an important class 1 GPCR, the AT receptor [59]. Additionally, we showed that another intracellular domain of the receptor (i3 loop) also contains a sequence for which CaM and βγ compete. We further demonstrated that the sequence requirements for CaM and βγ binding to both sites in the AT_1A_ receptor overlap, but are not identical. Regardless of whether CaM prevents binding of βγ, or hastens its displacement, this process can clearly modulate GPCR signal transduction.

Overall, the present study suggests that CaM may function as a regulator at interface between AT_1A_ receptor and G proteins by targeting G protein βγ subunit interacting with the receptor.
